# More than a “touch of the flu”: a response to Mayrhuber et al’s ““with fever it’s the real flu I would say”: laypersons’ perception of common cold and influenza and their differences - a qualitative study in Austria, Belgium and Croatia”

**DOI:** 10.1186/s12879-019-4437-x

**Published:** 2019-10-30

**Authors:** Annemarie Jutel

**Affiliations:** Victoria University of Wellington (NZ), Wellington, New Zealand

**Keywords:** Influenza, Vaccination, Diagnosis

## Abstract

This short reply contests two assumptions made by the authors of Mayrhuber et al’s. “With fever it’s the real flu I would say.” The first is that there is influenza can be reliably defined by a medical case definition. The second is that this small qualitative study can be generalisable. However, it does underline the important point that technical diagnostic terms may be used on different registers by a variety of actors in the medical setting.

Elisabeth Anne-Sophie Mayrhuber and her colleagues have delved into the fascinating matter of influenza [1]. Indeed, there’s probably not a more familiar pathogenic concept, if not disease. People talk about “the ‘flu” with great familiarity and oft-times randomly, attributing all manner of ailments to the flu: “stomach flu” for GI upset, “a touch of the flu” for under the weather, and “man flu” for the presumed male complainer, not really ill at all.

But their article takes for granted two important assumptions that it should not have. The first is that the lay person’s version should be measured against the biomedical concepts. Our work on self-diagnosis of influenza revealed that people (be they lay or health professionals) whose reported symptoms aligned with the case definition of influenza were no more likely have had influenza than those who did not [2]. And this possibly should not have surprised us. As Fabrice Carrat’s team revealed in their metaanalysis, over 30% of individuals inoculated with influenza were asymptomatic even while actively shedding virus [3]. What did surprise us, however, is that the symptoms most likely to correlate with accurate self-diagnosis were runny nose and fatigue. What one should take from this is that flu is not necessarily what medicine thinks it is, so, verifying whether someone without medical education reels off features of a case definition of influenza is not necessarily helpful to diagnosis.

And, the authors should not forget that “flu” is rarely confirmed as influenza in general practice. It is explained as “flu,” and charted as “influenza like illness,” but the presence of the virus is not used to diagnose. The benign and self-limiting influenza infection may or may not conform to the symptom profiles that we attribute to it. Influenza infection is widely variable in its acuity and in its presentation [4], and not surprisingly, doctors, as much as lay people, resort to defining flu as being like we have always said it is, when we don’t really always know [5].

The second assumption is that if lay people understand influenza better, we can improve vaccination coverage. Influenza and vaccination are different subjects and need to be differently explored. Concluding that by understanding a disease, a subject will necessarily want a vaccination is a leap of faith.

Qualitative research provides a deeply textured understanding of the problems faced by groups and individuals, and is indeed, useful to understanding cultural practices and beliefs. However, the information revealed by three sets of 30 individuals in different countries cannot be generalised or used, as these authors claim, as a way of increasing uptake of vaccination. On the other hand, using the material gleaned from this qualitative study, the authors could certainly devise and implement further research to determine how generalisable these views may be to the broader populations they wish to enlist in vaccination campaigns.

However, what this paper, and many like it underline, is that in any technical setting (like, in this case, medicine) lay people and technicians may use the same words, but in different registers [6, 7]. Doctors and patients alike talk about “flu” as if everyone were on the same page, when possibly the only thing they really agree on is that they are dealing about a systemic; non-specific; and hopefully, self-limiting illness [8].

## Data Availability

not applicable
