# Supplementary material for: More than a “touch of the flu”: a response to Mayrhuber et al’s ““with fever it’s the real flu I would say”: laypersons’ perception of common cold and influenza and their differences - a qualitative study in Austria, Belgium and Croatia”
Source: BMC Infect Dis. 2019 Oct 30;19:921. doi: 10.1186/s12879-019-4437-x (PMC6820963; doi:10.1186/s12879-019-4437-x)
Supplement: Related texts

# Reply: Response to “more than a “touch of the flu”: a response to Mayrhuber et al’s “with fever it’s the real flu I would say”: laypersons’ perception of common cold and influenza and their differences - a qualitative study in Austria, Belgium and Croatia”

**Authors:** Wim Peersman, Nina van de Kraats, Goranka Petricek, Asja Ćosić Diviak, Silvia Wojczewski, Kathryn Hoffmann

We thank Jutel A. for her interest in our work and the addition of interesting aspects and literature to our publication [1]. We agree that our study underlines the importance of respecting that technical diagnostic terms are used on different registers by a variety of actors in the medical setting [6, 7].

The objective of our study was to gain a deeper understanding of individuals in Austria, Belgium and Croatia regarding their reasoning and concept in terms of common cold, influenza and its differences as well as possible cross-country differences. This is what we have elaborated and described throughout the publication.

We are in line with the comment that influenza cannot be reliably defined by a medical case definition [2, 4], which we also stated in our publication: *“[…] Influenza is mainly recognized as starting with onset of a sudden high fever, whereas a common cold more commonly begins with coryza or a sore throat* [9, 10]*. Although from a microbiological point of view the two diseases are clearly different from each other as distinct viruses are involved* [9, 11, 12]*, it may be difficult to differentiate the diseases for lay persons because they share several symptoms such as fever, cough, or limb ache. This overlap has been observed to increase the risk of confusion not only in lay persons but also in clinicians* [9, 11, 13]*. […]”* However, besides this challenge it is important to gain a better understanding of laypersons’ reasoning and concepts of diseases to be able to pick up the individuals from their own starting points to increase communication and health literacy.

Surely, it is not enough to raise the lay person knowledge regarding influenza to improve health literacy or preventive measures like vaccination. Therefore, we discussed in addition a variety of measures like awareness activities, involvement of GPs, rapid tests as well as the use of timely epidemiological data [1]. Additionally, we agree with Jutel A. that the views collected with this sample cannot be generalized to the broader population. Namely, although the participants came from a broad range of socioeconomic backgrounds, we cannot assume that other themes would not arise in other localities and cultural groups. We clearly discussed the limitations of this qualitative study and mentioned among others the possibility for selection bias.

We welcome further research efforts to elaborate the topic further including a broader population.
